# Parameters of Blood Count and Tumor Markers in Patients with Borderline Ovarian Tumors: A Retrospective Analysis and Relation to Staging

**DOI:** 10.5402/2012/947831

**Published:** 2012-04-17

**Authors:** Rosekeila Simões Nomelini, Taísa Morete da Silva, Beatriz Martins Tavares Murta, Eddie Fernando Candido Murta

**Affiliations:** ^1^Discipline of Gynecology and Obstetrics, Oncological Research Institute (IPON), Federal University of Triângulo Mineiro, 38025-440 Uberaba, MG, Brazil; ^2^Discipline of Pharmacology, Federal University of Triângulo Mineiro, 38025-440 Uberaba, MG, Brazil

## Abstract

The aim of this paper was to evaluate the parameters of blood count and tumor markers in borderline ovarian tumors. We evaluated 21 patients who had confirmed histopathologic diagnosis of borderline ovarian tumor. We recorded age, parity, tumor type, stage of cancer, serum levels of tumor markers (CA-125, CA-15.3, CA-19.9, CEA, AFP), and the parameters of blood count, fasting glucose, disease-free survival and overall. The patients were divided into two groups, stage IA (*n* = 13) and stage IB-IIIC (*n* = 8). The unpaired *t*-test and Fisher's exact test were used, with *P* values of less than 0.05 being considered to indicate statistical significance. Levels of red blood cells, hematocrit, and hemoglobin were significantly higher in stage IA when compared with stage IB-IIIC (*P* < 0.05). The levels of tumor marker CEA had a tendency to be higher in the group stage IB-IIIC (0.08). Abnormal levels of CEA and CA-19.9 were found more frequently in stages IB-IIIC. Therefore, parameters of blood count, CEA, and CA-19.9 should be targeted for further research in identifying prognostic factors in borderline tumors.

## 1. Introduction

Borderline ovarian tumors were recognized as clinical and pathological entity by the International Federation of Gynecology and Obstetrics (FIGO) in 1972 and comprise about 15 to 20% of all malignant ovarian tumors [1, 2]. Histologically, the borderline tumors are defined by the presence of nuclear atypia, epithelial stratification, mitotic activity, and absence of stromal invasion [3, 4]. 

The most common histologic types are serous and mucinous tumors. They are usually staged according to FIGO but have a much better prognosis than invasive carcinomas [5]. Borderline ovarian tumors are also described as tumors with low malignant potential [6]. However, a subset of these tumors may progress and be lethal [7], making the diagnosis and treatment in cases of borderline tumors be still a challenge.

Borderline ovarian tumors are diagnosed primarily in young women who want to preserve their reproductive life and, therefore, it is important to define clinical and histological criteria for patients at high risk of obscuring the prognosis [8].

Studies of other molecular markers were not yet as reliable predictors of biological behavior; however, there is hope that future studies of genetics and molecular biology of tumors will provide the insertion of useful laboratory tests. Other prognosis factors were DNA-ploidy, histologic type, and age of patients [9].

Although patients have an excellent prognosis, recurrence risk remains in some cases. It was shown that the surgical procedure, the rupture of the cyst, the staging, the presence of microinvasion, and peritoneal implants were the five independent prognostic factors affecting the recurrence of these neoplasms [6, 7, 9, 10]. Emphasis should be provided in those patients with high-risk factors and prevention strategies should be taken to prevent its progression.

 The aim of this study was to evaluate the parameters of blood count and tumor markers in preoperative staging of borderline ovarian tumors.

## 2. Materials and Methods

 A retrospective study was conducted in the Departments of Special Pathology and Discipline of Obstetrics and Gynecology of UFTM. We evaluated 21 patients in the Pelvic Mass Clinic in the Discipline of Obstetrics and Gynecology/Oncology Research Institute (IPON) who were underwent surgical treatment according to predetermined criteria [11, 12] and had confirmed histopathologic diagnosis of borderline ovarian tumor.

 We recorded age, parity, tumor type, stage of cancer, type of surgery, rupture of the cyst, chemotherapy, serum levels of tumor markers (CA-125, CA-15.3, CA-19.9, CEA, AFP), and the parameters of blood count (hemoglobin, WBC, platelets), fasting glucose, disease-free survival, and overall. The patients were divided into two groups, stage IA (*n* = 13) and stage IB-IIIC (*n* = 8). Regarding the histologic type, tumors were divided into two groups, serous and nonserous. The normal values for tumor markers were AFP ≤ 12.1 ng/mL, CA 125 < 35 U/mL, CA15.3 < 25 U/mL, CA 19.9 < 39 U/mL, and CEA ≤ 5.5 ng/mL.

### 2.1. Statistical Analysis

Data were analyzed using GraphPad Instat software. Values were expressed as means ± standard deviations (SDs). Unpaired *t*-test was used to compare mean values of laboratory parameters between stages I and IB-IIIC and the mean values of laboratory parameters between the nonserous and serous group. Fisher's exact test was used to evaluate normal and abnormal levels of tumor markers in relation to groups of stages and histological type. The significance level was less than 0.05.

 This research was approved by the Research Ethics Committee of Federal University of Triângulo Mineiro.

## 3. Results

 We evaluated 21 patients with borderline ovarian tumor. The average age of patients was 50.05 ± 17.61 years. The average parity was 2.4 ± 2.04 children. Thirteen patients (61.9%) underwent bilateral salpingoophorectomy, hysterectomy, omentectomy, peritoneal washing, and multiple biopsies, and eight (38.1%) underwent unilateral or bilateral salpingoophorectomy. Thirteen patients (61.9%) were in stage IA, 2 (9.52%) in stage IB, 3 (14.3%) in stage IC, 2 (9.52%) in stage IIIA, and one (4.76%) in stage IIIC. Ten tumors (47.6%) were mucinous, eight (38.1%) were serous, one (4.75%) was endometrioid, one (4.75%) was a Brenner tumor, and one (4.75%) was mucinous with benign Brenner. Fifteen (71.4%) did not receive chemotherapy, and 6 (28.6%) did. One (4.76%) patient died and 7 (33.33%) were lost to follow up. In 17 (81%) patients there was no rupture of the cyst and ruptured by 19%.

 In Table 1, patients were divided into two groups, one with stage IA (*n* = 13) and the other with stage IB-IIIC (*n* = 8), and the laboratory parameters were compared. Levels of red blood cells, hematocrit, and hemoglobin were significantly higher in stage IA when compared with the group stage IB-IIIC (*P* < 0.03, *P* < 0.01, and *P* < 0.02, resp.). The levels of tumor marker CEA had a tendency to be higher in the group stage IB-IIIC, when compared to the group stage IA (0.08).  Table 2 compares the levels of tumor markers among nonserous and serous subtypes, no statistically significant difference being demonstrated.

 Comparing the normal and abnormal values of tumor markers in relation to the stages (Table 3), abnormal values of CA 19.9 and CEA were found more frequently in the group stages IB-IIIC (*P* = 0.0357 and *P* = 0.0006, resp.). Comparing the normal and abnormal values of tumor markers in relation to the histological types (serous or nonserous), no statistically significant difference was demonstrated.

## 4. Discussion

The treatment of borderline ovarian tumors was similar to their invasive counterparts for a long time. However, in view of the good prognosis, its occurrence in a younger age group, and the development of less invasive techniques, the question that can be asked is whether a conservative treatment is justified in these cases. The goal in treating cases of borderline ovarian tumor is the definition of patients with poor prognosis and risk factors for recurrence and therefore requires more aggressive therapy. Research could help identify borderline tumors with poor prognosis [13].

Although borderline ovarian tumors have an excellent prognosis, they are not free of risk of recurrence. Kokawa et al. (2009) showed that less advanced staging which is the absence of residual tumor, peritoneal implants and ovarian stromal involvement, and negative peritoneal cytology were significantly associated with improved overall survival. There are insufficient data to support a role for aggressive surgery and adjuvant chemotherapy-prolonged survival [14]. Chang et al. (2008) showed that the presence of invasive implants and micropapillary pattern were important prognostic factors in patients with serous borderline ovarian tumors [15]. Genetic abnormalities may better delineate the relationship between borderline tumors and carcinomas and lead to a unifying hypothesis as to the origin of these important ovarian lesions [7].

Some studies have shown changes in laboratory parameters of blood count and glucose related to the prognosis of malignant neoplasms. A higher frequency of changes in leukocytes in peripheral blood was found in patients with advanced cancer of the cervix, and neutrophilia could be an indicator of tumor invasiveness [16]. Another study showed that patients with cervical neoplasias with poor prognosis had higher plasma glucose levels than those with less aggressive lesions [17]. In ovarian malignancies, hemoglobin levels before and during chemotherapy may be important prognostic factors [18–20], and the hemoglobin concentration had a prognostic factor in oral squamous carcinoma [21].

However most studies relating to blood count and prognosis of cancer were performed in malignancies, and no similar studies were performed in borderline ovarian tumors. In our study, levels of red blood cells, hematocrit, and hemoglobin were significantly higher in stage IA when compared with the group stage IB-IIIC.

Ovarian cancer patients had haematological anomalies (haemoglobin and haematocrit levels were significantly lower and the platelet count was higher, and lower values for lymphocytes) compared to patients with benign ovarian tumours [22]. Some studies have shown a possible relationship with IL-6 levels of hemoglobin and platelets in malignant disease. High pretreatment platelet counts and low pretreatment hemoglobin levels can be negative prognostic factors in patients with ovarian cancer; there was observed the relationship of cystic fluid IL-6 levels with platelet counts and hemoglobin levels [23]. Another study demonstrated that anaemia in advanced stages of ovarian cancer may be related to the levels of IL-6 [24]. Therefore there can be a possible causative role of tumor-derived IL-6 and abnormal values in hemoglobin and platelet levels, which have been recognized as prognostic factors. In our study, there were no significant changes in glucose levels, leukocytes, and platelets. The small amount of patients studied because of the low frequency of borderline tumors may be a limiting factor.

In patients with borderline ovarian tumors, histological type mucinous, preoperative CA 19.9 was more frequently elevated than CA 125 or CEA, and abnormal levels of tumor markers predicted recurrence in follow-up [25]. Our data showed no significant difference in tumor markers between nonserous or serous group, perhaps by the small number of patients when groups are stratified.

Studies with a larger number of patients who underwent surgery for borderline ovarian tumors are needed to assess its importance as a prognostic factor. The CA-125 levels are may be higher in patients with borderline tumors in more advanced stages. Kolwijck et al. demonstrated preoperative serum CA-125 levels significantly higher for patients with advanced stage compared with patients with stage I in borderline ovarian tumors [26]. Wu et al. showed that stages II and III, only excision of the cyst (conserving ovarian tissue) and higher preoperative serum CA-125 were independent variables predictive of recurrence in these tumors [27]. Høgdall et al. showed that a shorter disease-specific survival for patients with 30% or higher CEA expression in the tumour tissue and the highest CEA expression compared with no expression was found to be an independent prognostic factor for mucinous ovarian cancer in Danish patients [28]. In a preoperative comparison of serum tumor markers, CA 125 and CA 19.9 levels were significantly higher in IC stages than those in IA stages [29]. CA 125 levels in our study were higher in stage IB-IIIC, but there was no statistical significance. The CEA levels tended to be higher also in these stagings. On the other hand, we found two abnormal tumor markers (CA 19.9 and CEA) more frequently in stages IB-IIIC compared with stage IA.

Some questions are still challenging. What are the high-risk patients? In patients who received adjuvant chemotherapy, would that provide benefits? When surgery could be performed preserving fertility in patients taking low risk of relapse? To our knowledge, this is the first study of literature that relates blood count and staging of borderline ovarian tumors. 

## 5. Conclusions

Red blood cell count, hemoglobin, and hematocrit were significantly higher in borderline ovarian tumors on initial staging (IA), and CEA levels tended to be higher in stage IB-IIIC. Abnormal values of CA 19.9 and CEA were found more frequently in the stages IB-IIIC. These laboratory parameters should be targeted for further research in identifying prognostic factors in borderline tumors, guiding the management of these cases and indicating for each patient the right treatment, so that it is less aggressive and may preserve fertility in women over young, but do not leave these patients at risk of an unfavorable evolution. Further studies with larger numbers of patients are needed to elucidate the role of laboratory parameters in the prognosis of borderline ovarian tumor.

## Figures and Tables

**Table 1:** Laboratory parameters (mean ± standard deviation) in stage IA (*n* = 13) and IB-IIIC (*n* = 8) borderline ovarian tumors.

	Stage IA	Stages IB-IIIC
Red blood cells (×10^6^/mm^3^)	4.78 ± 0.47*	4.25 ± 0.5
Hematocrit (%)	41.9 ± 3.6**	36.91 ± 5.13
Haemoglobin (g/100 mL)	14.08 ± 1.3***	12.37 ± 1.9
Leucocytes (/mm^3^)	7, 740 ± 2, 607.1	8,162.5 ± 5, 246.5
Platelets (/mm^3^)	259,076.92 ± 58, 150	310,857.14 ± 88, 494
Fasting glucose (mg/dL)	100.2 ± 25.93	104.5 ± 28.05
CA-125 (U/mL)	60.47 ± 132.8	64.34 ± 59.43
CA-15.3 (U/mL)	41.12 ± 53.55	28.85 ± 17.84
CA-19.9 (U/mL)	12.95 ± 13.8	23.84 ± 36.35
CEA (U/mL)	2.43 ± 3.7	9.86 ± 12.48
AFP (U/mL)	1.43 ± 0.65	1.42 ± 0.56

Unpaired *t*-test. *,**,***respectively, *P* < 0.03, 0.01, 0.02, compared to stages IB-IIIC.

**Table 2:** Tumor markers (mean ± standard deviation) in serous histological subtypes (*n* = 8) and nonserous (*n* = 13) borderline ovarian tumors.

	Serous Mean ± standard deviation	Nonserous Mean ± standard deviation
CA-125 (U/mL)	44.87 ± 59.99	75.19 ± 136.71
CA-15.3 (U/mL)	53.66 ± 62.3	23.19 ± 11.31
CA-19.9 (U/mL)	7.01 ± 5.01	24.95 ± 29.38
CEA (U/mL)	1.31 ± 1.22	7.67 ± 10.31
AFP (U/mL)	1.74 ± 0.62	1.24 ± 0.54

Unpaired *t*-test; *P*: not significant.

**Table 3:** Increased tumour markers (*n*/total of cases) in relation to groups of stages.

	Stage IA	%	Stages IB-IIIC	%
CA-125 (≥35 U/mL)	2/10	20	4/6	66.7
CA-15.3 (≥25 U/mL)	4/10	40	2/6	33.3
CA-19.9 (≥39 U/mL)	1/10	10	4/6*	66.7
CEA (≥5.5 ng/mL)	1/11	9.1	6/6**	100
AFP (≥12.1 ng/mL)	0/11	0	0/5	0

Fisher's exact test. *, **, respectively, *P* < 0.0357 and 0.0006, compared to stage I.
